# Deletion of *Pdcd5* in mice led to the deficiency of placenta development and embryonic lethality

**DOI:** 10.1038/cddis.2017.124

**Published:** 2017-05-25

**Authors:** Ge Li, Chentong Xu, Xin Lin, Liujing Qu, Dan Xia, Beiqi Hongdu, Yan Xia, Xiaokun Wang, Yaxin Lou, Qihua He, Dalong Ma, Yingyu Chen

**Affiliations:** 1 Department of Immunology, Peking University School of Basic Medical Science, No. 38 Xueyuan Road, Beijing 100191, China; 2 Key Laboratory of Medical Immunology, Ministry of Health, Peking University Health Sciences Center, No. 38 Xueyuan Road, Beijing 100191, China; 3 The Clinical Laboratory, Affiliated Hospital of Inner Mongolia Medical University, No. 1 Tongdao North Street, Hohhot, Inner Mongolia 010050, China; 4 Center for Human Disease Genomics, Peking University, No. 38 Xueyuan Road, Beijing 100191, China; 5 Medical and Healthy Analytical Center, Peking University, No. 38 Xueyuan Road, Beijing 100191, China

## Abstract

Programmed cell death 5 (PDCD5) is an apoptosis promoter molecule that displays multiple biological activities. However, the function of PDCD5 *in vivo* has not yet been investigated. Here, we generated a *Pdcd5* knockout mouse model to study the physiological role of PDCD5 *in vivo*. Knockout of the *Pdcd5* gene resulted in embryonic lethality at mid-gestation. Histopathological analysis revealed dysplasia in both the LZs and JZs in *Pdcd5*^*–/–*^ placentas with defects in spongiotrophoblasts and trophoblast giant cells. Furthermore, *Pdcd5*^*–/–*^ embryos had impaired transplacental passage capacity. We also found that *Pdcd5*^*–/–*^ embryos exhibited cardiac abnormalities and defective liver development. The growth defect is linked to impaired placental development and may be caused by insufficient oxygen and nutrient transfer across the placenta. These findings were verified *in vitro* in *Pdcd5* knockout mouse embryonic fibroblasts, which showed increased apoptosis and G0/G1 phase cell cycle arrest. *Pdcd5* knockout decreased the Vegf and hepatocyte growth factor (Hgf) levels, downregulated the downstream Pik3ca–Akt–Mtor signal pathway and decreased cell survival. Collectively, our studies demonstrated that *Pdcd5* knockout in mouse embryos results in placental defects and embryonic lethality.

The *PDCD5* (programmed cell death 5) gene, formerly designated as *TFAR19* (TF-1 cell apoptosis-related gene-19), was first discovered in 1999.^1^ Existing studies on PDCD5 have revealed that its expression is downregulated in a variety of tumor tissues, and PDCD5 overexpression can promote apoptosis in different tumor cell types in response to various stimuli,^1, 2, 3^ as well as participate in other types of cell death such as paraptosis^4^ and autophagy.^5^ Studies have revealed that PDCD5 can interact with multiple molecules such as TIP60,^3^ TP53,^6, 7^ MDM2^6^ and HDAC3,^7^ and PDCD5 has been shown to play an important role in the TIP60/HDAC3-TP53 signaling pathway to promote apoptosis and G0/G1 phase cell cycle arrest. PDCD5 can also interact with CCT^8^ to inhibit *β*-tubulin folding and influence cell division and proliferation.

*PDCD5* expression in cells is finely regulated. Transcription factor NF-*κ*B p65 promotes the levels of *PDCD5* mRNA and transcriptional activity.^9^ OTU deubiquitinase 5 (OTUD5) and YY1-associated factor 2 (YAF2) interact with PDCD5 to enhance its stability and inhibit proteasome-dependent degradation.^10, 11^ In contrast, DNAJB1 enhances ubiquitination and degradation of PDCD5.^12^

Recently, we found that PDCD5 interacts with FOXP3, increases acetylation of FOXP3 in synergy with TIP60 and enhances the repressive function of FOXP3.^13^ In *PDCD5* transgenic (*PDCD5tg*) mice, *PDCD5* overexpression could alleviate the severity of experimentally induced autoimmune encephalomyelitis (EAE).^13^ In a rat collagen-induced arthritis model, rats that were treated with recombinant human PDCD5 protein significantly delayed the occurrence and reduced the severity of arthritis.^14^ Moreover, rhPDCD5 also exerted anti-inflammatory effects in EAE mice.^15^ Additionally, PDCD5 may also play an important role in influenza virus (H1N1) infection,^16^ cerebral ischemia/reperfusion injury^17^ and cardiac remodeling,^5^ indicating the diversity and complexity of its functions.

Thus far, however, the function of Pdcd5 *in vivo* remains totally unknown. Therefore, in the present study, we generated *Pdcd5* knockout (*Pdcd5*^*–/–*^) mice to study the physiological role of Pdcd5 *in vivo.*

## Results

### *Pdcd5* knockout in mice leads to embryonic lethality

**Figure 1:**
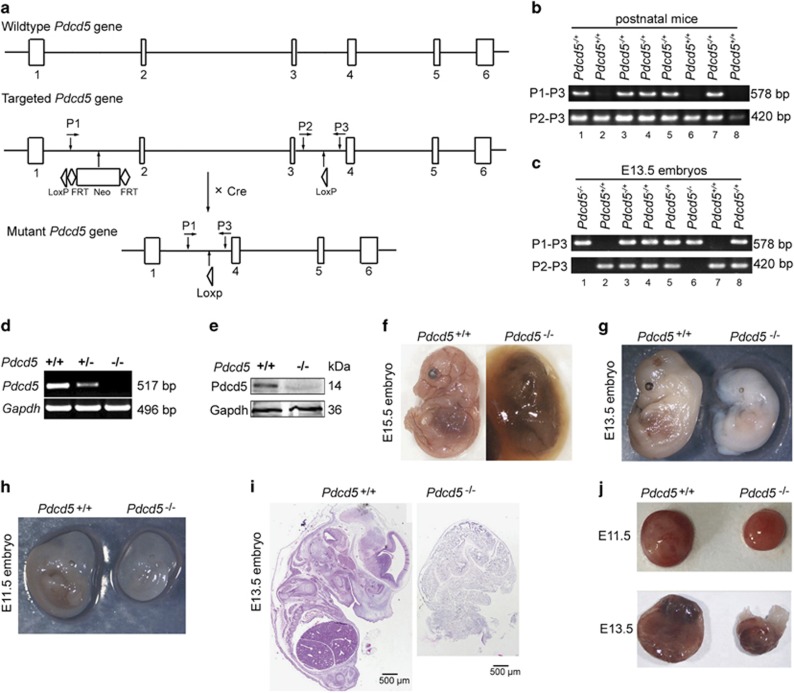
*Pdcd5* knockout mice exhibited intrauterine growth restriction and embryonic lethality. (**a**) Schematic representation of the mouse *Pdcd5* genomic structure, the LoxP-modified *Pdcd5* loci and the mutant *Pdcd5* structure. Boxes represent the six *Pdcd5* exons. The triangles represent the LoxP sites, and primers P1, P2 and P3 are indicated for PCR genotyping. (**b** and **c**) Representative PCR results for genotyping of postnatal mice (**b**) and E12.5 embryos (**c**) derived from intercrossing between *Pdcd5*^+/–^ mice. (**d**) The relative *Pdcd5* mRNA levels were analyzed in *Pdcd5*^*+/+*^, *Pdcd5*^*−/+*^ and *Pdcd5*^*–/–*^ embryos by RT-PCR. (**e**) The expression of Pdcd5 protein in *Pdcd5*^*+/+*^ and *Pdcd5*^*–/–*^embryos detected by western blot analysis. (**f–h**) Representative images of *Pdcd5*^*+/+*^ and *Pdcd5*^*–/–*^ embryos at E15.5 (**f**), E13.5 (**g**) and E11.5 (**h**). (**i**) *Pdcd5*^*+/+*^ and *Pdcd5*^*–/–*^embryos at E13.5 were analyzed by hematoxylin and eosin (H&E) staining. **(j**) Representative images of *Pdcd5*^*+/+*^ and *Pdcd5*^*–/–*^placentas at E11.5 and E13.5

Using *Zp3-Cre* transgenic mice, in which *Pdcd5* is exclusively expressed in growing oocytes under the control of the zona pellucida glycoprotein 3 promoter,^18^ we obtained heterozygous *Pdcd5* (*Pdcd5*^+/–^) mutant mice with deleted exons 2 and 3. Figure 1a is a schematic representation of the mouse *Pdcd5* genomic structure, the LoxP-modified *Pdcd5* loci and the mutant *Pdcd5* structure. Long-term observation revealed that *Pdcd5*^+/−^ mice appeared phenotypically normal, and the eldest mouse survived beyond 15 months of age without evidence of significant spontaneous disease. Theoretically, *Pdcd5*^*–/–*^ mice can be generated by the intercrossing of *Pdcd5*^+/−^ mice. However, genotyping of more than 100 pups identified no homozygous mice (Figure 1b), whereas *Pdcd5*^*+/+*^ and *Pdcd5*^+/−^pups had a Mendelian ratio of nearly 1:2 (Supplementary Table S1). These results indicated that pups homozygous for *Pdcd5*^*–/−*^ died *in utero*.

To identify the time of death, embryos derived from heterozygous intercrosses were genotyped at various stages of gestation. The results of the genotype and phenotype analysis are summarized in Supplementary Table S1. As shown in the table, no viable *Pdcd5*^*–/–*^ embryos were recovered after embryonic day 13.5 (E13.5). Representative PCR identification of mouse embryos at E12.5 is shown in Figure 1c. Results of the RT-PCR and western blot analyses demonstrated that normal *Pdcd5* mRNA and Pdcd5 protein were undetectable in *Pdcd5*^*–/–*^ embryos (Figures 1d and e). At E15.5, the *Pdcd5*^*–/–*^ embryos were obviously dead and resorbed (Figure 1f), and we could not detect any live *Pdcd5*^*−/−*^embryos at E15.5. Although visible homozygous embryos could still be detected at E13.5, they appeared soft and pale, and could not maintain their shape (Figure 1g). Histological analysis of embryos obtained at E13.5 showed that *Pdcd5*^*–/–*^ embryos had undergone complete autolysis, and none of the organs could be identified (Figure 1i), suggesting that the point of lethality for the homozygous embryos was approximately E13.5. At E11.5, *Pdcd5*^*–/–*^ embryos were also smaller and paler than *Pdcd5*^*+/+*^ embryos (Figure 1h). At E12.5 embryos, the architecture of mutant embryos appeared normal, but the overall shape of these embryos was smaller than that of the wild-type (WT) embryos (Supplementary Figure S1). These results showed that homozygous deletion of *Pdcd5* led to growth retardation and embryonic lethality at mid-gestation, suggesting that Pdcd5 is indispensable in fetal development.

### Impaired development of junctional and labyrinth zones in *Pdcd5*^*–/–*^ placentas

**Figure 2:**
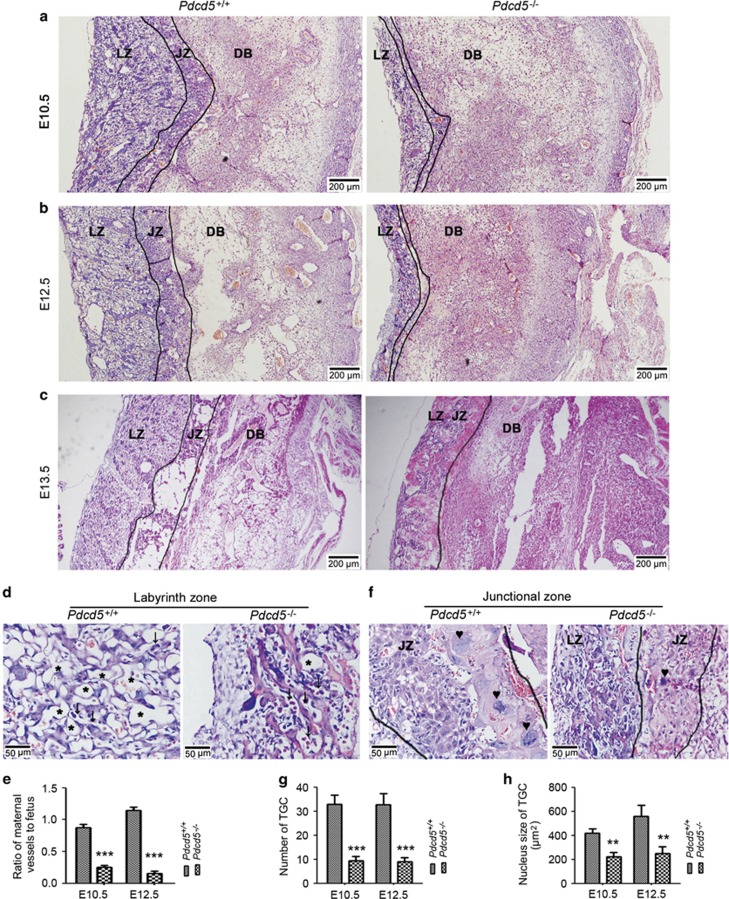
Developmental defects in the labyrinth and junctional zones in *Pdcd5*^*–/–*^ placentas. (**a–c**) H&E staining of *Pdcd5*^*+/+*^ and *Pdcd5*^*–/–*^ placentas at E10.5 (**a**), E12.5 (**b**) and E13.5 (**c**). The layers of the placental architecture are labeled: decidua basalis (DB), junctional zone (JZ) and labyrinth zone (LZ). (**d**) H&E staining of the LZ of *Pdcd5*^*+/+*^ and *Pdcd5*^*–/–*^ placentas at E12.5. Vessels were separated by erythrocytes of maternal blood sinuses (asterisks) and fetal vessels (arrows). (**e**) Histogram shows the ratio of maternal vessels/fetus from *Pdcd5*^*+/+*^ and *Pdcd5*^*–/–*^ LZ at E10.5 and E12.5. Three placentas from each genotype and five different sections from each placenta were analyzed. Data are means±S.D. ***P<0.001. (**f**) H&E staining of the JZ of *Pdcd5*^*+/+*^ and *Pdcd5*^*–/–*^ placentas at E12.5. (**g** and **h**) Histogram shows the number (**g**) and nucleus size (**h**) of TGC from *Pdcd5*^*+/+*^ and *Pdcd5*^*–/–*^ JZ at E10.5 and E12.5. Three placentas from each genotype and five different sections from each placenta were analyzed. Data are means±S.D. ***P<0.001, **P<0.01

**Figure 3:**
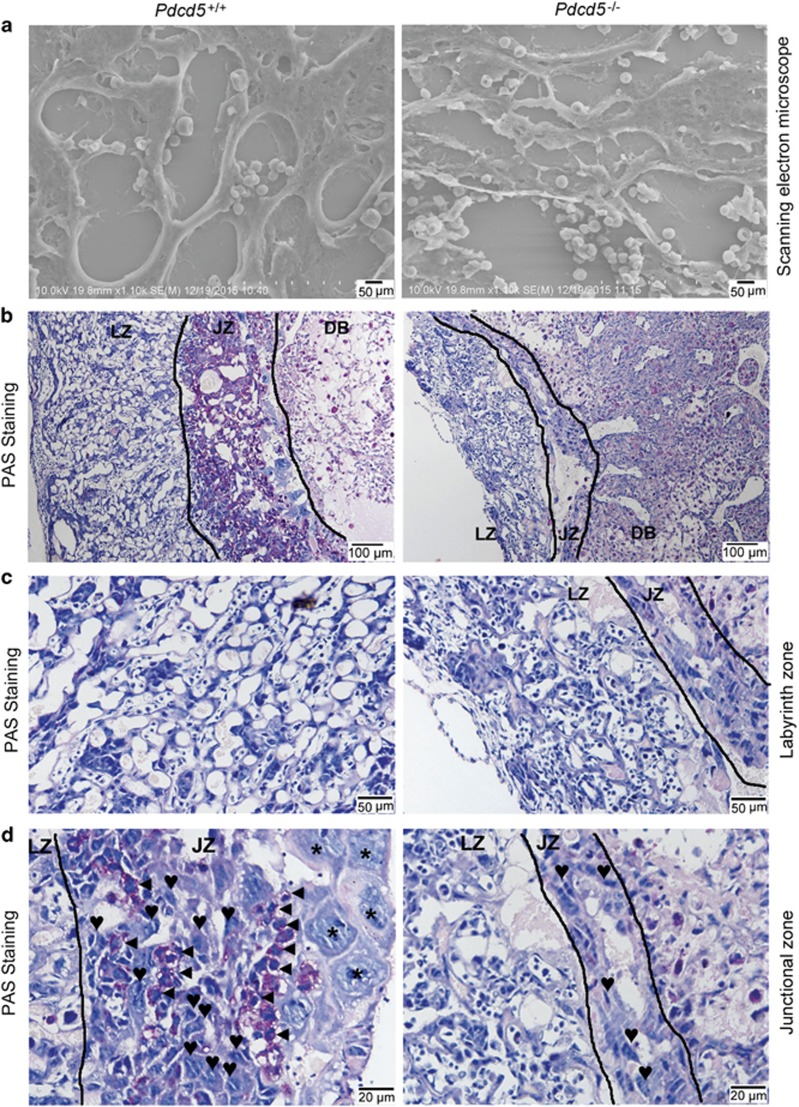
Analysis of *Pdcd5*^*+/+*^ and *Pdcd5*^*–/–*^ placentas by scanning electron microscopy and PAS staining. (**a**) Scanning electron microscope analysis of the labyrinth zone in *Pdcd5*^*+/+*^ and *Pdcd5*^*–/–*^ placentas at E12.5 (× 10 000). (**b**) Periodic acid/Schiff (PAS) staining of *Pdcd5*^*+/+*^ and *Pdcd5*^*–/–*^ placentas at E12.5. The layers of the placental architecture are labeled: decidua basalis (DB), junctional zone (JZ) and labyrinth zone (LZ). (**c** and **d**) PAS staining in *Pdcd5*^*+/+*^ and *Pdcd5*^*−/−*^ LZ and JZ at E12.5. Trophoblasts are marked as trophoblast giant cells (asterisks), glycogen trophoblasts (triangles) and spongiotrophoblasts (hearts)

Gross examination of the placenta revealed that the *Pdcd5*^*–/–*^ placentas were smaller than the *Pdcd5*^*+/+*^ placentas (Figure 1j); therefore, we performed histological analysis of the placental tissues. As shown in Figures 2a–c, *Pdcd5*^*+/+*^ placentas showed normal architecture with three compartments: maternal decidua basalis (DB), junctional zone (JZ) and labyrinth zone (LZ) where nutrient transfer occurs between the maternal and fetal blood spaces.^19^ However, LZ and JZ exhibited a drastic reduction in thickness in the *Pdcd5*^*–/–*^placenta (Figures 2a–c, left panel). At E13.5, the mutant placentas showed disordered structure, hemorrhage and necrosis compared with *Pdcd5*^*+/+*^ placentas (Figure 2c). At a higher magnification, we observed that the number of invading maternal blood sinuses in *Pdcd5*^*–/–*^ LZ was strongly decreased compared with that in normal LZ (Figure 2d). The mutant LZ did not have the highly vascularized organization and appeared to be in a compact state. There was no obvious difference in the number of fetal blood vessels (identified by the nucleated embryonic blood cells within them) between *Pdcd5*^*–/–*^ and *Pdcd5*^*+/+*^ LZ. Accordingly, the ratio of maternal blood vessels to fetal blood vessels was reduced in mutant LZ (Figure 2e). Ultrastructure analysis of the LZ (Figure 3a) revealed a disordered alignment of blood vessels in the *Pdcd5*^*–/–*^ LZ, indicating that the LZ was markedly underdeveloped in *Pdcd5*^*–/–*^ placentas.

The JZ is composed of spongiotrophoblasts (SpTs), glycogen trophoblasts (GlyTCs) and trophoblast giant cells (TGCs). The TGCs are located at the uterine trophoblastic border and play a role in endocrine function. As illustrated in Figures 2f–h, the number and size of TGCs were lower in the *Pdcd5*^–/–^ JZ than in the *Pdcd5*^*+/+*^ JZ. Similarly, the number of SpTs and GlyTCs was also significantly decreased in the homozygous mutant JZ. The paraffin-sliced sections of the placentas were further stained with periodic acid/Schiff (PAS) at E12.5 (Figures 3b and c). The boundary between the JZ and LZ was more clearly visible with PAS staining than with H&E staining (Figure 3b), and the JZ and LZ were both thinner in *Pdcd5*^*−/−*^ placentas than in *Pdcd5*^+/+^ placentas. At the same time, visualization at a higher magnification revealed a rich network structure in the *Pdcd5*^+/+^ LZ with tubular cavity (maternal blood vessels) (Figure 3c, left). However, only few maternal blood vessels were present in *Pdcd5*^–/–^ LZ, which appeared very compact (Figure 3c, right). These findings were consistent with the results observed by H&E staining (Figures 2d and e). PAS-positive GlyTCs in *Pdcd5*^+/+^ JZ were stained magenta with small and dense nuclei and vacuoles in the cytoplasm. SpTs and TGCs are not stained with PAS and appear gray in the cytoplasm with large nuclei (Figure 3d, left). However, the area occupied by GlyTCs and TGCs had almost disappeared in the *Pdcd5*^–/–^JZ, and only PAS-negative SpTs were present (Figure 3d, right). Collectively, these data confirmed that the development of the LZ and JZ was significantly impaired by the absence of *Pdcd5*.

### Placental transport is impaired in *Pdcd5*^*–/–*^ mice

**Figure 4:**
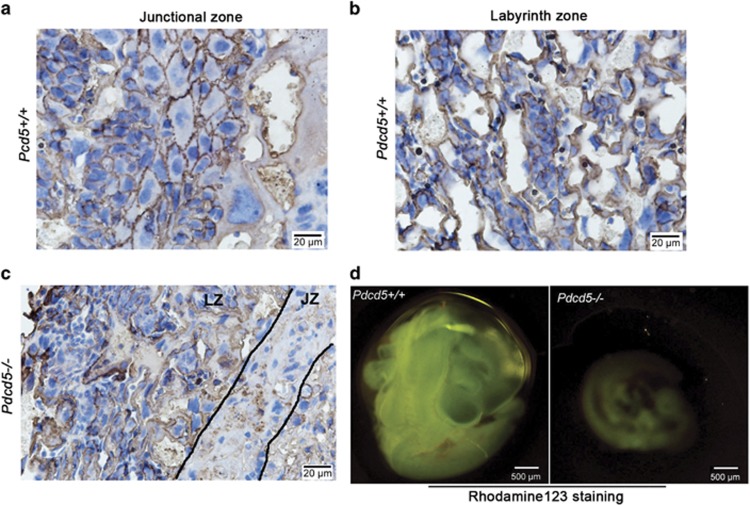
Impaired placental transport in *Pdcd5*^*–/–*^ placentas. (**a–c**) Glut-1 staining of the junctional zone (**a**) and LZ (**b**) in *Pdcd5*^*+/+*^at E12.5, and of the junctional and labyrinth zones in *Pdcd5*^*–/–*^(**c**). (**d**) Transplacental passage of rhodamine 123 dye. One hour after tail vein injection of rhodamine 123, passive passage of the dye from the mother to the embryo was detected in *Pdcd5*^*+/+*^ and *Pdcd5*^*–/–*^ embryos at E10.5

GLUT-1 is the predominant form expressed in the placenta during early pregnancy.^20^ Abundant GLUT-1 expression is crucial to transplacental glucose transfer via the placenta. Next, we investigated Glut-1 protein expression in the placentas at E12.5. Membrane-expressed Glut-1 was detected in both SpTs and GlyTCs but not in the TGCs of the *Pdcd5*^+/+^ JZ (Figure 4a). On the contrary, Glut-1 expression was almost absent in the JZ of *Pdcd5*^*–/–*^ placentas because of defects in SpTs and GlyTCs (Figure 4c). There was no significant difference in the Glut-1 distribution at LZs between the two groups (Figures 4b and c). As glucose is a major nutrient passing through the placenta from the mother to the growing embryos, it is reasonable to suggest that the underdeveloped placenta may be associated with a reduction in the glucose transport capacity.

We next measured transplacental passage by injecting pregnant mice with the fluorescent dye rhodamine 123. As shown in Figure 4d, *Pdcd5*^–/–^ embryos showed significantly less fluorescence than *Pdcd5*^+/+^ embryos. We also observed that *Pdcd5*^–/–^ embryos were smaller than *Pdcd5*^+/+^ embryos.

To determine whether *Pdcd5*^–/–^ placentas displayed abnormal proliferation or apoptosis, the placental specimens were stained with Ki-67. The number of Ki-67-positive cells was undetectable in the *Pdcd5*^*–/–*^ JZ (Supplementary Figure S2b) compared with the *Pdcd5*^+/+^ JZ at E12.5 (Supplementary Figure S2a). The Ki-67 signal did not significantly differ between the *Pdcd5*^*–/–*^ and *Pdcd5*^+/+^ LZs. We further assessed cell apoptosis by terminal deoxynucleotidyl transferase dUTP nick-end labeling (TUNEL) assay and cleaved caspase-3 staining. There were no significant differences between the *Pdcd5*^*–/–*^ and *Pdcd5*^*+/+*^ placentas (Supplementary Figures S2c–e). Taken together, these results suggested that loss of *Pdcd5* is associated with defects in placental development, accompanied by inhibition of cell proliferation.

### Vegf, Vegfr-2 and Pecam-1 levels were decreased in the *Pdcd5*^–/–^ LZ

The placenta is a vital organ facilitating the exchange of gases and nutrients between the fetus and the mother, and its physiological functions are closely related with vascular formation. Establishing a vascular network and blood circulation determine placental function, and protect the growth and development of the fetus. As the establishment of the vascular network in *Pdcd5*^–/–^ placentas was defective (Figures 2 and 3), we attempted to analyze the regulatory mechanism of placental vasculogenesis.

**Figure 5:**
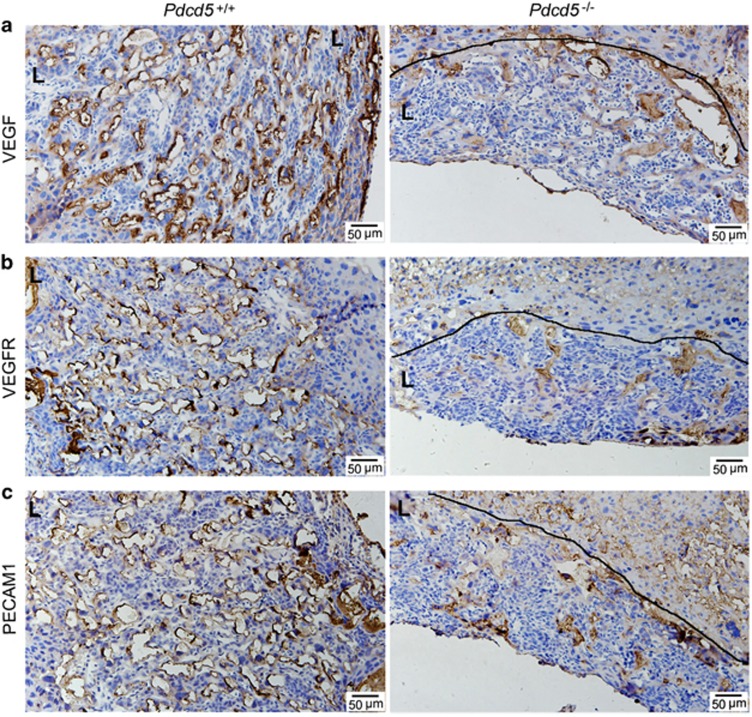
Angiogenesis was defective in the placental labyrinth zone of *Pdcd5* knockout. (**a–c**) Immunohistochemical staining of Vegf (**a**), Vegfr-2 (**b**) and Pecam-1 (**c**) in the labyrinth zones of *Pdcd5*^*+/+*^ and *Pdcd5*^*–/–*^ placentas at E12.5

Placental angiogenesis is regulated by vascular endothelial growth factor (Vegf) and its high-affinity receptor tyrosine kinases Vegfr. The results revealed that Vegf is highly expressed in maternal vascular endothelial cells in the *Pdcd5*^+/+^ LZ (Figure 5a). Vegfr-2, the primary Vegf receptor, is essential for normal endothelial proliferation and vascular formation. We found that the distribution of Vegfr-2 was consistent with that of Vegf in *Pdcd5*^*+/+*^ placentas (Figure 5b, left)**.** On the contrary, Vegf and Vegfr-2-expressing cells were barely visible in the *Pdcd5*^–/–^ LZ (Figures 5a and b).

PECAM-1 mediates endothelial integrity and other functions, including vascular permeability, regulation of bioavailability of nitric oxide and angiogenesis. Pecam-1 exhibited strong signaling in the *Pdcd5*^+/+^ LZ zone but not in the *Pdcd5*^*−/−*^ placenta (Figure 5c). The Vegf, Vegfr-2 and Pecam-1 expression levels in fetal blood vessels were negative in the LZ of both *Pdcd5*^–/–^ and *Pdcd5*^*+/+*^ placentas. These data suggest that *Pdcd5* knockout strongly impaired endothelial integrity or function in mouse placental samples.

### Expression of Pdcd5 in mouse placentas, embryonic livers and hearts

The embryonic lethality observed in *Pdcd5*^*–/–*^ embryos indicated that Pdcd5 is indispensable for fetal development. We next analyzed the expression of Pdcd5 in WT placentas. As shown in Supplementary Figure S3, Pdcd5 was detected in both the cytoplasm and nucleus of the endothelial cells and cytotrophoblasts in the LZ (Supplementary Figure S3a). Pdcd5 was expressed in the nucleus of TGCs in the JZ at moderate levels (Supplementary Figure S3b). The lower immunoreactivity of Pdcd5 was detected in GlyTCs but not in SpTs (Supplementary Figure S3b). The DB cells expressed high or moderate levels of Pdcd5 in both the cytoplasm and the nucleus (Supplementary Figure S3c). The results of qRT-PCR revealed that the *Pdcd5* mRNA levels were significantly lower in *Pdcd5*^*–/–*^ placentas than in WT placentas, but completely absent in *Pdcd5*^*–/–*^ embryos (Supplementary Figure S3d).

The levels of *Pdcd5* mRNA were also detected by *in-situ* hybridization in E12.5 WT placenta. As shown in Supplementary Figures S3e–g, *Pdcd5* mRNA was expressed at low or moderate levels in the LZs and JZs. In particular, the *Pdcd5* mRNA signal was strong in the DB. We simultaneously analyzed Pdcd5 expression in WT embryonic livers and hearts at E12.5. *Pdcd5* mRNA and protein levels were undetectable in embryonic livers (Supplementary Figures S4a and b, left panels**)**, and low levels of *Pdcd5* mRNA and protein were expressed in embryonic hearts (Supplementary Figures 4a and b, right panels). It should be pointed out that *Pdcd5* mRNA levels were significantly higher in placentas than in embryonic livers and hearts.

### Injured livers and hearts in *Pdcd5* knockout mice

**Figure 6:**
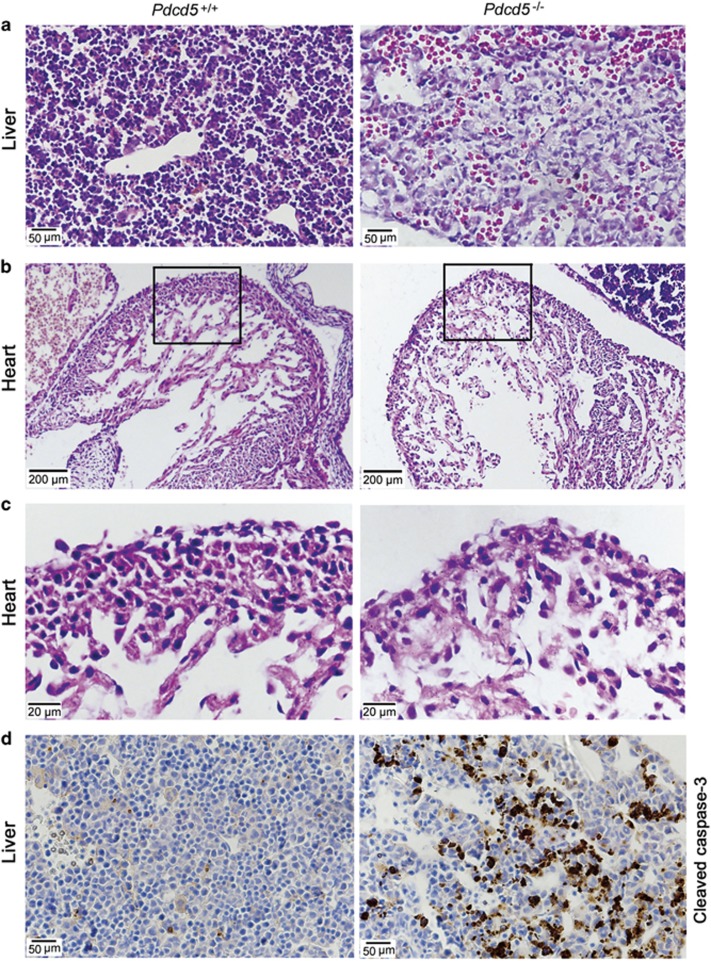
Damaged livers and hearts in *Pdcd5*^*–/–*^ embryos at E12.5. (**a**) The livers from *Pdcd5*^*+/+*^ or *Pdcd5*^*–/–*^ embryos were analyzed by H&E staining. (**b–c**) The hearts from *Pdcd5*^*+/+*^ or *Pdcd5*^*–/–*^ embryos were analyzed by H&E staining, magnified by 100 × (**b**) and 400 × (**c**). (**d**) The levels of cleaved caspase-3 protein in *Pdcd5*^*+/+*^ or *Pdcd5*^*–/–*^ livers were detected by immunohistochemical analysis

Embryonic development depends on successful placental maturation as well as cardiovascular maturation. Cardiovascular insufficiency is another major cause of embryonic lethality. Histological analysis of *Pdcd5*^*–/–*^ embryos revealed defects in liver development. At E12.5, parenchymal hepatocytes became denser and formed hepatic lobules in the *Pdcd5*^*+/+*^ liver, while *Pdcd5*^*–/–*^ livers displayed reduced cellularity and increased empty spaces, indicative of a relatively lower density of parenchymal hepatocytes (Figure 6a). At the same time, blood vessels including sinusoids were dilated, and numerous immature nucleated erythrocytes were observed in the *Pdcd5*^*–/–*^ liver (Figure 6a, right), exhibiting hemorrhage. Cell apoptosis assay revealed strong signals of cleaved caspase-3 in *Pdcd5*^*–/–*^ hepatocytes (Figure 6d).

Considering the core function of heart in circulation, morphogenetic defects in the heart can severely influence embryonic development. As shown in Figures 6b and c, the ventricular wall of the heart was thinner in *Pdcd5*^*–/–*^ embryos than in their *Pdcd5*^*+/+*^ counterparts; however, the architecture in both the groups was normal. At a higher magnification, the number of myocardial cells was visibly lower in the ventricular wall of *Pdcd5*^*–/–*^ embryos relative to that of *Pdcd5*^*+/+*^ embryos (Figure 6c). The atrial wall and papillary muscles of the *Pdcd5*^*–/–*^ and *Pdcd5*^*+/+*^ embryos did not show obvious differences. Next, we investigated whether the thin ventricular wall in *Pdcd5*^*–/–*^ heart was related to the increased apoptosis. Caspase-3 expression was not detected in both *Pdcd5*^*–/–*^ and *Pdcd5*^*+/+*^ hearts (Supplementary Figure S5), indicating that the defect in the *Pdcd5*^*–/–*^heart might have no direct relationship with cell apoptosis. In view of the increased Pdcd5 expression in *Pdcd5*^+/+^ embryonic liver and heart at E14.5 (Supplementary Figure S4c), which occurred in *Pdcd5*^*−/−*^ embryos around the time of death at E13.5 (Figures 1g and i), we considered that the major cause of embryonic lethality in *Pdcd5*-deficient mice may be placental failure. The abnormal phenomena observed in *Pdcd5*^–/–^ embryonic livers and hearts may probably be indirect consequences of placental failure.

### *Pdcd5*^*–/–*^ MEF cells exhibited growth arrest and apoptosis

**Figure 7:**
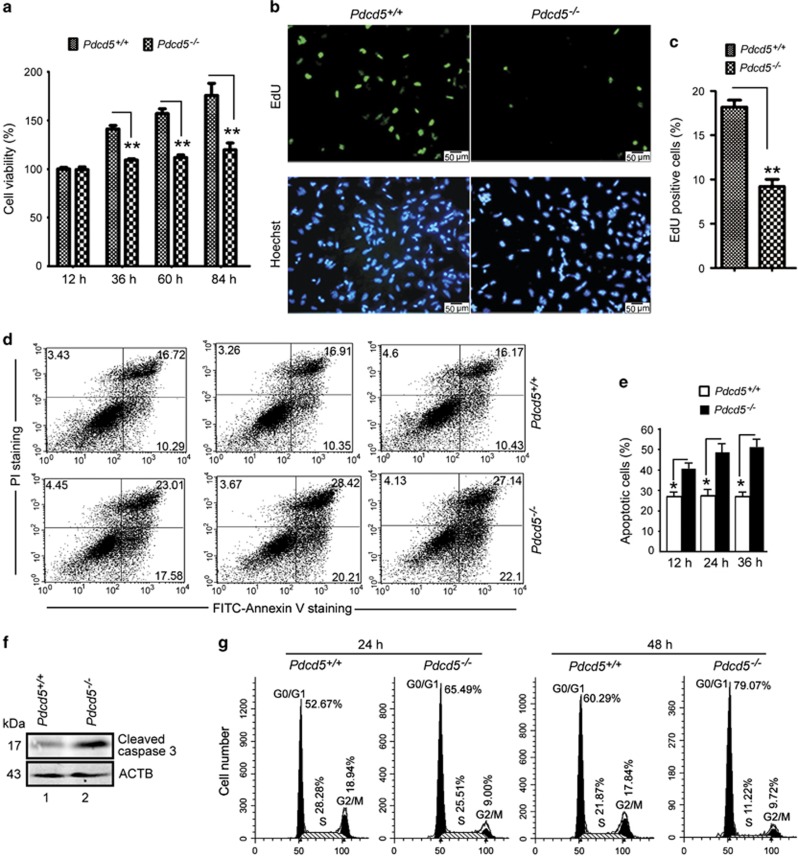
*Pdcd5*^*–/–*^ MEFs showed decreased proliferation, increased apoptosis and cell cycle arrest. (**a**) Cell viability was measured in *Pdcd5*^*+/+*^ and *Pdcd5*^*−/−*^ MEF cells (E10.5) at indicated time using the CCK-8 assay. Data are means±S.D. of the results from three independent experiments (**P<0.01). (**b**) *Pdcd5*^*+/+*^ and *Pdcd5*^*−/−*^ MEF cells at E10.5 were plated in glass slides and treated with EdU for 4 h, and then analyzed by immunofluorescence. Nuclei were stained with Hoechst 33342. Representative fluorescence microscopy images are shown. (**c**) Treated as (**b**), the ratio of EdU-positive cells to the total number of cells was counted in 10 visual fields and measured by the Student’s *t-*test. Data are means±S.D. of the results from three independent experiments (**P<0.01). (**d**) *Pdcd5*^*+/+*^ and *Pdcd5*^*−/−*^ MEF cells at E10.5 were cultured for 12, 24 and 36 h, and then analyzed by Annexin V/propidium iodide (PI) staining and flow cytometry. (**e**) The cleaved caspase-3 levels in *Pdcd5*^*+/+*^ and *Pdcd5*^*−/−*^ MEF cells at E10.5 were detected by western blot analysis. (**f**) *Pdcd5*^*+/+*^ and *Pdcd5*^*−/−*^ MEF cells at E10.5 were cultured for 24 or 48 h, and then analyzed by PI staining and flow cytometry

Based on the above findings, we further studied the biological activity of *Pdcd5*^*–/–*^ mice embryonic fibroblasts (MEFs). At E10.5 or E11.5, *Pdcd5*^*–/–*^ MEFs failed to adhere to the plate after separation 8 h up to 24 h, unlike *Pdcd5*^*+/+*^ MEFs (Supplementary Figures S6a and b). The size of *Pdcd5*^*–/–*^ MEFs appeared smaller than *Pdcd5*^*+/+*^ MEFs. Results of the flow cytometry study further confirmed this phenotype (Supplementary Figures S6c). To verify this finding, we analyzed cell viability and growth kinetics using the CCK-8 and EdU (5-ethynyl-2′-deoxyuridine) incorporation assays. The results revealed that *Pdcd5*^*–/–*^ MEFs were significantly less viable than *Pdcd5*^*+/+*^ MEFs (Figure 7a). The EdU-positive cells in the *Pdcd5*^*–/–*^ MEFs were significantly lower than in the *Pdcd5*^+/+^ MEFs (Figures 7b and c), indicating that *Pdcd5* knockdown inhibited cell proliferation.

We next determined cell apoptosis by measuring the phosphatidylserine externalization in MEFs. The results revealed a significantly higher number of apoptotic cells in *Pdcd5*^*–/–*^ MEFs in a time-dependent manner (Figure 7d). This phenotype accompanied by caspase-3 activation (Figure 7e). At the same time, *Pdcd5*^*–/–*^ MEFs showed a G0/G1 phase arrest at both 24 and 48 h (Figure 7f). Together, these data suggest that *Pdcd5* knockdown could suppress cell growth, promote apoptosis and induce cell cycle arrest in MEFs.

### Loss of *Pdcd5* inhibited Hgf-mediated Pik3ca/Akt–Mtor signaling

**Figure 8:**
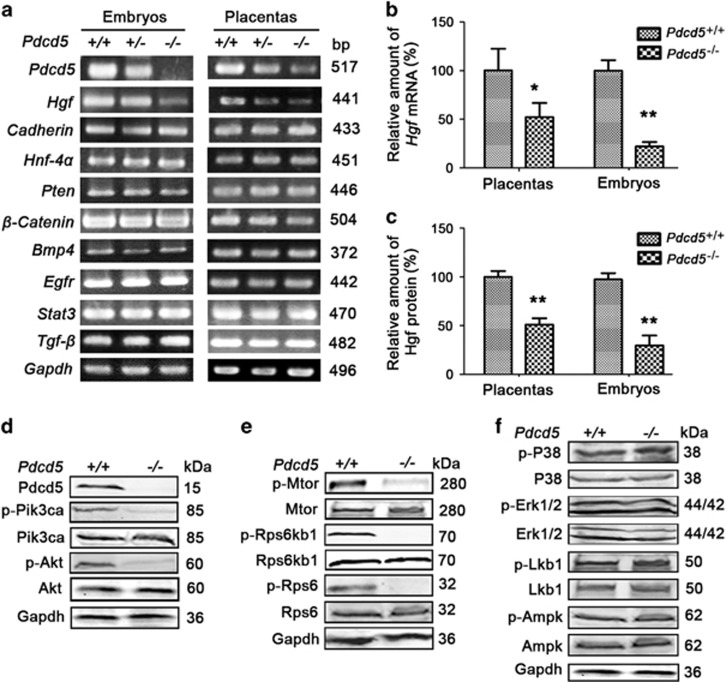
The Hgf–Pik3ca–Mtor signal pathway was inhibited after *Pdcd5* knockout. (**a–c**) The *Hgf* mRNA and protein levels in different embryos and placentas at E11.5 were detected by RT-PCR (**a**), qRT-PCR (**b**) and ELISA (**c**). (**d–f**) Proteins from *Pdcd5*^*+/+*^ or *Pdcd5*^*–/–*^ embryos at E11.5 were extracted and subjected to western blot analysis using the indicated antibodies

To investigate the mechanism by which *Pdcd5* knockout leads to embryonic lethality, we performed RT-PCR in embryos, placentas and MEFs to detect various signal pathway molecules. Among the detected genes, the *Stat3, Tgf-β, Cadherin, Pten, β-catenin, Bmp4, Egfr* and *Hnf-4α* transcript levels showed no obvious change in the mutant embryos, placentas and MEF cells (Figure 8a and Supplementary Figure S7a). Surprisingly, *Hgf* mRNA was distinctly decreased in *Pdcd5*^*–/–*^ relative to that in the *Pdcd5*^*+/+*^ and *Pdcd5*^*–/+*^. The decreased transcription of *Hgf* in *Pdcd5*^*–/–*^ embryos and placentas was further confirmed by quantitative RT-PCR (Figure 8b). The low levels of Hgf protein expressed in *Pdcd5*^*–/–*^ placentas and embryos were confirmed by ELISA assay in tissue lysates (Figure 8c).

We then analyzed the changes in the signaling molecules downstream of Hgf to determine the signaling pathways that are affected by *Pdcd5* knockdown. There was a significant decrease in Pik3ca (Y458) and Akt (S473) phosphorylation in the *Pdcd5*^*−/−*^ embryos at E12.5 (Figure 8d). Simultaneously, the phosphorylation levels of Mtor at S2448, Rps6kb1 at T389 and Rps6 at S235/236 were also decreased in the *Pdcd5*^*–/–*^ embryos (Figure 8e). Similar data were obtained in *Pdcd5*^*–/–*^ MEFs (Supplementary Figure S7b). Additionally, loss of *Pdcd5* had no significant influence on the phosphorylation of P38 Mapk, Erk 1/2, Lkb1 and Ampk (Figure 8f). Collectively, these results suggested that the embryonic lethality caused by *Pdcd5* loss plays a role in the inhibition of the Hgf-regulated Pik3ca/Akt–Mtor signaling pathway.

## Discussion

The results of the present study reveal the unique and unexpected role of Pdcd5 as a key regulator in the process of embryonic development. Genetic disruption of *Pdcd5* resulted in impaired placental development, damaged placental angiogenesis and reduced nutrient supply to the fetus, consequently injuring the livers and hearts of embryos, leading to fetal growth retardation and death. The Pdcd5 levels were significantly higher in the placenta than in the embryonic liver and heart, suggesting that Pdcd5 may play a more important role in the placenta than in the fetus during embryonic development. Currently, it remains unknown whether the heart and liver defects were the primary reason for the observed embryonic lethal phenotype. Increase in the Pdcd5 expression occurred in *Pdcd5*^+/+^ embryonic livers and hearts at E14.5 (Supplementary Figure S4c), which lagged behind the increase in *Pdcd5*^*−/−*^ embryos coinciding with embryonic death at E13.5 (Figures 1g and i). Based on these findings, we speculate that the abnormal phenomena in *Pdcd5*^–/–^ embryonic liver and heart could be an indirect consequence of placental failure. In the present study, our findings demonstrate for the first time that Pdcd5 is a vital protein whose function cannot be compensated by other molecules in fetal development. Considering the pleiotropic functions ascribed to Pdcd5,^21^ it is likely that a multitude of factors contribute to embryonic lethality in mouse embryos lacking *Pdcd5*.

Several molecules that interact with Pdcd5, such as Vhl, Tip60, Hdac3 and Ck2, can cause embryonic lethality in the corresponding gene knockout mice. Histone acetyltransferase Tip60 and histone deacetylase Hdac3 interact with Pdcd5 to regulate Tp53 activation.^3, 7^ Ck2 mediates Pdcd5 phosphorylation and nuclear translocation. Vhl also participates in Pdcd5 location.^21^ *Tip60* gene ablation causes early embryonic lethality at the eight-cell blastocyst stage (E3.5) and homozygous-null *Tip60*^–/–^ mice do not survive up to the post-gastrulation stages of development.^22^ In another study, *Hdac3*^–/–^ mice were reported to have died before E9.5 due to defects in gastrulation.^23^ *Ck2*-deficient embryos stopped development at the blastocyst stage and were resorbed at E7.5.^24^ Similarly, *Vhl* KO mice have been found to exhibit embryonic lethality as a result of abnormal placental development.^25^ *Vhl*-deficient mice died *in utero* from E10.5 to E12.5, following defective vasculogenesis and hemorrhage of the placental LZ. The Vegf levels in the labyrinth trophoblasts were greatly reduced in *Vhl*-deficient placentas relative to normal placentas;^25^ this is similar to the results obtained in *Pdcd5*^*–/–*^ placentas. As a multitude of PDCD5-interacting molecules also trigger embryonic lethality when their expression levels are affected, *Pdcd5* may function during embryonic development by regulating the above-mentioned molecules or in similar pathways.

The placenta is a highly vascularized tissue that develops during early gestation to facilitate the circulation of blood, oxygen, glucose and nutrients between the mother and the fetus. Placental failure was a major contributing factor to embryonic lethality. Trophoblast cells of the JZ are located at the trophoblast border of the uterus and can migrate into the DB and perform endocrine function. The LZ is the main site of contact between the fetal and maternal circulation, consisting of a highly branched fetal vascular network.^19^ *Pdcd5* deletion affected placental vascularization (Figures 2, 3, 4, 5). Such alterations could impair embryonic growth and viability as the highly vascular LZ is the site of maternal–fetal oxygen and nutrient exchange.

TGCs are the first cell type to terminally differentiate during embryogenesis and are of vital importance for implantation and modulation of post-implantation placentation. TGCs mediate blastocyst attachment and invasion into the uterine epithelium, regulate decidualization of the uterus and anastomose with maternal blood spaces to form the transient yolk sac placenta. TGCs secrete a wide array of hormones and paracrine factors, including Vegf, Hgf and prolactin-related cytokines, to target the maternal physiological systems for proper maternal adaptations to pregnancy and the fetal–maternal interface to ensure vasculature remodeling.^26, 27^ TGCs also secrete urokinase-type plasminogen activator (uPA),^28^ which is a classical HGF activator to mediate pro-HGF cleavage and maturation.^29^ In *Pdcd5* KO placentas, the number and size of TGCs had significantly decreased, resulting in a reduction in the Vegf and Hgf levels in placentas and embryos. Vegf-deficient mice underwent embryonic lethality before E8.5 due to the lack of vasculogenesis and blood island development.^30^ *Hgf*^–/–^ mice embryos were found to exhibit reduced liver and placenta sizes and fewer hepatocytes and labyrinthine trophoblast cells relative to their wild-type counterparts. Moreover, the labyrinth region of the placenta was poorly developed, although the number of trophoblast cells in the JZ appeared normal.^31, 32, 33^ Based on these results, we can conclude that the decreased Vegf and Hgf levels in *Pdcd5*^–/–^ mice may negatively regulate the expression and phosphorylation of Pik3ca/AKT–Mtor signaling, which occurred in the present study. This may play a contributory role in the defective vascularization and embryonic lethality.

Thus far, the roles of SpTs and GlyTCs in the JZ of mouse placenta remain unknown. It is thought that GlyTCs participate in glucose metabolism and utilize glucose as a source of energy.^34^ Glucose is one of the most important substances transferred from the maternal blood to the fetal circulation across the placenta, and it is a primary energy source for the fetus. Glucose transport to the fetus across the placenta takes place via glucose transporters, and the Glut-1 glucose transporter appears to be the rate-limiting step in transplacental transport.^35^ The number of SpTs and GlyTCs was obviously reduced in *Pdcd5*^–/–^ placentas than in *Pdcd5*^+/+^ placentas, and the Glut-1 expression level was lower in the former than in the latter. In particular, the rhodamine 123 transport assay revealed that the placental nutrient transport function was decreased in *Pdcd5*^–/–^ mice. Together with the insufficient placental vascularization, the lack of energy and nutrition in *Pdcd5*^–/–^ mice was responsible for the abnormal embryogenesis, and ultimately death.

Many tumors have been shown to have reduced Pdcd5 levels, implicating its potential role as a tumor suppressor.^21^ However, heterozygous deletion of *Pdcd5* in mice did not lead to the development of any tumors until 1 year of age (unpublished data). Perhaps the one remaining WT allele in heterozygous mice is sufficient to maintain the potential tumor suppressor function of *Pdcd5*. For many tumor suppressor genes, germline single-allele loss in combination with stochastic somatic loss has been shown to result in an increased incidence of tumors in certain organs. For example, total deletion of tumor suppressor gene *Brac1* results in embryonic lethality, whereas mice heterozygous for *Brac1* survived and did not develop tumors. Notably, introduction of a *TP53*-null allele significantly enhances mammary gland tumor formation in *Brca1* conditional KO mice.^36^ Therefore, it is possible that another allele might function synergistically to enhance cancer development in *Pdcd5* heterozygous mice. Future studies using *Pdcd5* conditional knockout mice crossed with other mice tumor models will be necessary to identify if the embryonic lethality of *Pdcd5* homozygous deletion can be overcome and to clarify the potential role of *Pdcd5* as a tumor suppressor *in vivo.*

## Materials and Methods

### Generation of *Pdcd5* knockout mice

Mice were maintained on a C57BL/6 genetic background. Mice harboring a floxed conditional knockout cassette of *Pdcd5* were constructed by the Model Animal Research Center of Nanjing University (Nanjing, China). A FRT-Neo-FRT-LoxP cassette was inserted into intron 1 of the *Pdcd5* gene and a LoxP site was inserted into intron 3. Mice carrying the floxed *Pdcd5* allele (*Pdcd5*^*+/flox*^) were mated to mice expressing Cre recombinase under control of the *Zp3* promoter (*Zp3-Cre*). The Cre recombinase can be expressed in the growing oocyte of *Zp3-Cre* transgenic mice prior to the completion of the first meiotic division.^18^ To generate heterozygous *Pdcd5* knockout (*Pdcd5*^+/−^) mice, these *Pdcd5*^*+/flox*^:*Zp3-Cre* or *Pdcd5*^*flox/flox*^: *Zp3-Cre* mice were further mated with WT male mice. All mice were bred at the Experimental Animal Center, Peking University Health Sciences Center (Beijing, China) under a 12-h light/dark cycle. All mice were given free access to water and standard mouse chow. All protocols used within this study were approved by the Animal Care and Use Committee of the University.

### Genotype analysis by PCR

*Pdcd5* genotyping was performed by PCR using DNA isolated from the yolk sacs of embryos or from tails of postnatal mice. Tissues were soaked in 100 *μ*l of 25 mM NaOH and 0.2 mM EDTA lysis buffer, heated for 30 min at 95 °C, and neutralized using 100 *μ*l of 40 mM Tris-HCl (pH 5.5). The following primer sequences were used for *Pdcd5* genotyping: P1, 5′-GGACTCCAGAGATGGTGCTCAG-3′ P2, 5′-TTGTCATGGTCATGGGAGCT-3′ and P3, 5′-TTTTCAGGCTTTACAAGTGC-3′. The following PCR cycling conditions were used: denaturation at 95 °C for 3 min; 35 cycles of amplification at 95 °C for 30 s, 58 °C for 30 s and 72 °C for 30 s; and a final extension step at 72 °C for 5 min.

### Antibodies and reagents

Rabbit anti-PDCD5 polyclonal antibodies used here have been described before.^6^ The following other antibodies were used in this study: anti-Ki-67 (ab16667), anti-HGF (ab83760), anti-GLUT-1 (ab115730), anti-VEGFA (ab52917) and anti-cleaved caspase-3 (ab13847) were purchased from Abcam (Cambridge, UK); and anti-PECAM-1 (SAB4502167), rhodamine 123 (R8004) and Hoechst 33342 (14533) were purchased from Sigma Aldrich (St. Louis, MO, USA). Signaling pathway-related antibodies were purchased from Cell Signaling Technology (Danvers, MA, USA): anti-PIK3CA p85 (4292 s), anti-phospho- PIK3CA p85 (Try458, 4228 s), anti-AKT (9272 s), anti-phospho-AKT (Ser473, 4060 s), anti-Mtor (2983 s), anti-phospho-Mtor (Ser2448, 2971 s), anti-RPS6KB1 (2708 s), anti-phospho-RPS6KB1 (Thr389, 9234 s), anti-RPS6 (2217 s), anti-phospho-RPS6 (Ser235/236, 4858 s), anti-ERK (4695p), anti-phospho-ERK (Thr202/Tyr204, 4370 s), anti-AMPK (2532 s), anti-phospho-AMPK (Thr172, 2535 s), anti-LKB1 (3047 s), ani-phospho-LKB1 (Ser428, 3482 s), anti-p38 MAPK (9212p) and anti-phospho-p38 MAPK (Thr180/Tyr182, 4511p). Secondary antibodies included DyLight 800/DyLight 680-conjugated IgG against mouse (610-145-002/610-144-002, Rockland, Philadelphia, PA, USA) or rabbit (611-145-002/611-144-002, Rockland, Philadelphia, PA, USA) antibodies. RIPA lysis buffer (50 mM Tris-HCl, pH 7.4, 150 mM NaCl, 1% NP-40; P0013D) was purchased from Beyotime (Shanghai, China).

### RT-PCR and quantitative real-time PCR (qRT-PCR)

Total RNA was prepared from embryos or placentas using TRIzol reagent (15596; Invitrogen, Carlsbad, CA, USA). cDNA was synthesized using ReverAid First Strand cDNA Synthesis Kit (K1622; Invitrogen). mRNA expression was analyzed by RT-PCR or qRT-PCR and normalized to the expression of the *Gapdh* housekeeping gene. RT-PCR and qRT-PCR assays were performed in triplicate for each sample. Supplementary Table S2 lists the primers used for RT-PCR and qRT-PCR.

### Western blot

Proteins from the embryos, placentas and MEFs were extracted using RIPA buffer containing phosphatase and protease inhibitor cocktail (04693116001/04906837001; Roche Diagnostics, Berlin, Germany). The cell lysates (80 *μ*g) were resolved by sodium dodecyl sulfate-polyacrylamide gel electrophoresis (SDS-PAGE), transferred to nitrocellulose membrane (Whatman, Buckinghamshire, UK) and probed by the indicated antibodies. The protein bands were visualized using DyLight 800/DyLight 680-conjugated secondary antibodies, and an infrared fluorescence image was obtained using an Odyssey infrared imaging system (LI-COR Biosciences, Lincoln, NE, USA).

### Histological and immunohistochemical analysis

The embryos and placentas were fixed in formalin overnight and embedded in paraffin. For histopathological analysis, 4-*μ*m sections were stained with hematoxylin and eosin (H&E) using standard procedures. A PAS staining kit (H0133; Harvey, Beijing, China) was used to stain glycogen-containing cells.

For immunohistochemical analysis, the embryo and placenta sections were dewaxed and rehydrated in xylol and a graded alcohol series containing decreasing concentrations of ethanol, followed by heat-induced antigen retrieval in citrate buffer. The sections were then treated by 3% H_2_O_2_ to quench endogenous peroxidase activity, and incubated with 5% goat serum. After incubation with the primary antibodies and biotinylated secondary antibody (PV-9000; Origen, Rockville, MD, USA), the color reaction was developed using 3,3′-diaminobenzidine (ZLI-9031; Origen).

### Scanning electron microscopy

The placentas were fixed in formalin overnight and embedded in paraffin. For the scanning electron microscopy, 4-*μ*m sections were placed on monocrystalline silicon piece, and dewaxed in xylol and ethanol. The sections were desalinated by repeated washing in deionized water and dehydrated in ethanol. The samples were coated with gold spray, and the sections were observed under a scanning electron microscope (S4800, JEOL, Tokyo, Japan).

### Generation of MEF cells and cell culture

MEF cells were isolated from E10.5 or E11.5 mice embryos. Embryos were harvested, washed with phosphate-buffered saline, minced and trypsinized (0.25% trypsin) for 30 min at 25 °C. The cell suspensions were cultured in 10-cm dishes containing 10 ml of Dulbecco’s modified Eagle’s medium containing 10% fetal bovine serum and 1% penicillin/streptomycin in a CO_2_ incubator at 37 °C. The following studies of MEF cells were used in their second generation.

### CCK-8 assay

Cell viability was determined with the CCK-8 reduction assay. MEF cells were seeded in a 96-well plate (3 × 10^3^ cells/well) and incubated for 12, 36, 60 and 84 h. Then, 10 *μ*l of CCK-8 solution was added to each well, and the cells were further incubated at 37 °C for 4 h. Absorbance was measured at 450 nm, and a reference was recorded at 630 nm with a microplate reader (Thermo, Waltham, MA, USA).

### EdU incorporation assay

The EdU incorporation assay was performed using an EdU Imaging Kit (C10337; Invitrogen). MEF cells were seeded in the wells of a 24-well plate (1 × 10^4^ cells per well) for 24 h, incubated with EdU at 37 °C for 4 h, fixed with 4% paraformaldehyde, permeabilized with 0.2% Triton X-100 and stained to observe EdU staining. The nuclei were stained by Hoechst 33342 for 5 min. Fluorescence signals were detected under a fluorescence microscope (BX53; Olympus, Miyazaki, Japan).

### Apoptosis assay

Cell apoptosis was detected using an Annexin V-fluorescein isothiocyanate (FITC) Apoptosis Detection Kit (Beijing Biosea Biotechnology Co., Ltd, Beijing, China) according to the manufacturer’s instructions. MEFs were first cultured in six-well plates (3 × 10^5^ cells per well) for 12, 24 and 36 h, and collected and stained with Annexin V-FITC and propidium iodide (PI). The cells were then run through a FACSCalibur flow cytometer (BD Biosciences, Franklin Lakes, NJ, USA) to determine the percentage of apoptotic cells.

Tissue apoptosis was detected using an *In Situ* Cell Death Detection Kit (1168479591; Roche Diagnostics) by a TUNEL assay. Frozen placenta sections from both *Pdcd5*^*−/−*^ and *Pdcd5*^+/+^ mice at E12.5 were treated and stained according to the manufacturer’s directions.

### Cell cycle analysis

MEF cells were seeded in a six-well plate (3 × 10^5^ per well) and cultured for 24 or 48 h. The cells were then fixed with 70% ethanol at 4 °C overnight, treated with 100 *μ*g/ml RNase A for 30 min at 37 °C, stained using PI in 0.2% Triton X-100, and analyzed with a FACSCalibur flow cytometer (BD Biosciences).

### Enzyme-linked immunosorbent assay (ELISA)

The Hgf in embyros and placentas were detected using a mouse ELISA Kit (ab100687; Abcam). The total protein was detected by a BCA protein assay kit (P0012; Beyotime). Data were presented as the ratio of Hgf protein level to the total protein level.

### Statistical analysis

Data were presented as the means±S.D. Differences between groups were analyzed using the Student’s *t-*test for continuous variables. Statistical significance in this study was set at *P<0.05, **P<0.01 and ***P<0.001. All reported *P*-values are two-sided. All analyses were performed with GraphPad Prism 5 (GraphPad Software, Inc, La Jolla, CA USA).
